# Second neoplasms associated with primary cutaneous lymphomas^☆^^☆☆^

**DOI:** 10.1016/j.abd.2018.12.004

**Published:** 2019-10-26

**Authors:** María Encarnación Gómez Sánchez, Maria Rodríguez Vázquez, Manuela Mollejo Villanueva

**Affiliations:** a Dermatology Service, Hospital General de Villarrobledo, Albacete, Spain; b Dermatology Service, Complejo Hospitalario Universitario de Albacete, Albacete, Spain; c Pathology Service, Hospital Virgen de la Salud, Complejo Hospitalario de Toledo, Toledo, Spain

*Dear Editor*,

Primary cutaneous lymphomas (PCL) are a diverse group of non-Hodgkin lymphomas (NHL) with initial presentation in the skin. Their annual incidence is estimated to be 1:100,000.^1^ Some studies have suggested that patients with PCL are at higher risk of developing secondary cancers compared with the general population.^2^, ^3^, ^4^

We conducted a retrospective cohort study of patients with clinical and histopathological diagnosis of PCL receiving care at a tertiary referral hospital in Albacete, Spain from January 1993 through May 2014 (health coverage area of 402,296 inhabitants). Clinical, laboratory, and histopathological data were evaluated, as well as the association of a second neoplasm. Patients were classified according to the WHO/EORTC 2005 and WHO 2008 classifications.^1^ The study was approved by the hospital ethics committee.

We assessed 95 patients with PCL; 58 (61.05%) were primary cutaneous T-cell lymphoma (PCTCL), 36 (37.89%) were primary cutaneous B-cell lymphoma (PCBCL), and one patient (1.05%) had blastic plasmacytoid dendritic cell neoplasm (BPDCN). Sixteen patients (16.84%) were found with one or more associated second neoplasm, ten in PCTCL (17.24%) and six in PCBCL (16.67%) (Figure 1, Figure 2). There were ten men (61.1%) and six women (37.9%). The patients corresponded to 62.5% T-linage (six men and four women) and 37.5% B-linage (four men and two women). The average age was 64.6 years (SD ± 17.88). There were in total 19 neoplasms. Four tumors (21.05%) were previous and 15 (78.94%) were subsequent to the diagnosis of lymphoma. Among these patients, eight (42.1%) new hematological neoplasms were found, all in the group of T-linage. One was systemic (one case of Hodgkin lymphoma) and seven cases had some other PCL: one case of primary cutaneous marginal zone lymphoma (PCMZL), four cases of lymphomatoid papulosis (LyP), and two cases of mycosis fungoides (MF). However, there were 11 (57.9%) solid tumors, four (36.6%) in PCTCL (epidermoid carcinoma of the tongue, colon adenocarcinoma, lung carcinoma, and lung metastases of unknown origin) and seven (63.63%) in PCBCL (lung carcinoma, hepatocellular carcinoma, pancreatic neuroendocrine tumor, gastric adenocarcinoma, and one patient who had had a urothelial tumor two years before the primary cutaneous follicle center lymphoma (PCFCL) and a subsequent metastasis of unknown origin in the liver). Three patients had more than one neoplasm: one with lung carcinoma and LyP after MF, another with colon adenocarcinoma and LyP after primary cutaneous anaplastic large-cell lymphoma (PCALCL), and a third patient with a urothelial tumor that had developed two years before the diagnosis of lymphoma and a hepatic metastases of unknown origin that was simultaneously detected in the extension study. Previous immunosuppressive therapy that could predispose to a second neoplasm was only found in two patients. In one case, the diagnosis was simultaneous, and in 13 patients, the second neoplasm developed between three months and 12 years after the diagnosis of PCL. The average time of second neoplasm onset was 2.73 years (SD ± 3.23), being 3.5 years (SD ± 3.42) in T-cell lineage and 0.43 years (SD ± 0.12) in B-cell-lineage lymphomas. The mean time of having a previous neoplasia was three years (SD ± 2.56).

**Figure 1:**
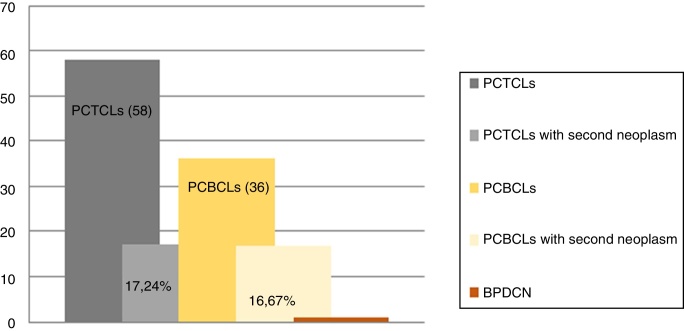
Primary cutaneous lymphoma and second neoplasms according to origin of T/B lineage. PCTCLs, primary cutaneous t-cell lymphoma; PCBC-L, primary cutaneous b-cell lymphoma; BPDCN, blastic plasmacytoid dendritic cell neoplasm.

**Figure 2:**
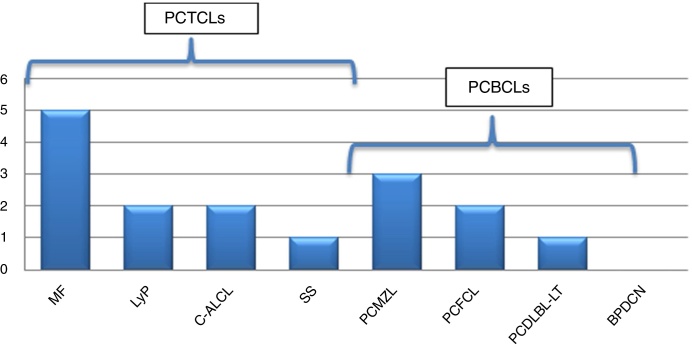
Frequency of second neoplasm according to subtypes of PCL. MF, mycosis fungoides; LyP, lymphomatoid papulosis; C-ALCL, cutaneous anaplastic large-cell lymphoma; SS, Sezary syndrome; PCMZL, primary cutaneous marginal zone lymphoma; PCFCL, primary cutaneous follicle center lymphoma; PCDLBL-TP, primary cutaneous diffuse large b-cell lymphoma; BPDCN, blastic plasmacytoid dendritic cell neoplasm.

Brownell et al.^2^ reported the largest study of risk of cancer in 672 patients with CTCL. They found that 16.7% of the patients suffered additional cancer similar to the present study's outcomes (17.24%) in a CTCL population. This and other previous studies have reported an increase of hematological and solid neoplasm in this group. The origin of solid neoplasm is quite variable. We found a slight increase of hematological neoplasms in PCTCL, as was observed by other authors,^2^, ^3^ as well as in lung cancer, but the greater number of solid neoplasms and the absence of hematological disease found in our PCBCL population is noteworthy. Chan et al.^4^ found a higher rate of second malignancies in PCBCL than the present group (25.5% *vs.* 16.67%) especially hematologic neoplasm and skin cancer. To our knowledge, this latter study and our report are the largest analyses of second malignancies in PCBCL ever reported. The risk of developing any second malignancy is greater within the first year following diagnosis.^2^ In our group, most of second tumors appeared in the first three years (2.73 ± 3.23). It should be considered that in one patient, the second tumor appeared 12 years following diagnosis.

In conclusion, skin lymphomas may result in patients with a reduced immunity and decreased cancer surveillance, resulting in a susceptibility to second tumors.^3^, ^4^, ^5^ We identified a high rate of second neoplasm in PCL. Thorough and lifelong medical surveillance could be necessary to ensure an early detection of second cancer, which usually determines patient prognosis. However, more studies are needed in this regard.

## Financial support

None declared.

## Author's contribution

María Encarnación Gómez Sánchez: Elaboration and writing of the manuscript; obtaining, analyzing and interpreting the data; intellectual participation in propaedeutic and/or therapeutic conduct of the cases studied; critical review of the literature.

Maria Rodríguez Vázquez: Approval of the final version of the manuscript; obtaining, analyzing and interpreting the data; effective participation in research orientation; critical review of the manuscript.

Manuela Mollejo Villanueva: Approval of the final version of the manuscript; effective participation in research orientation; critical review of the manuscript.

## Conflicts of interest

None declared.
